# Ganglioside Monosialic Acid Alleviates Peripheral Neuropathy Induced by Utidelone Plus Capecitabine in Metastatic Breast Cancer From a Phase III Clinical Trial

**DOI:** 10.3389/fonc.2020.524223

**Published:** 2020-10-09

**Authors:** Junnan Xu, Yan Wang, Cui Jiang, Hui Cao, Junhan Jiang, Binghe Xu, Tao Sun

**Affiliations:** ^1^Department of Medical Oncology, Liaoning Cancer Hospital & Institute, Cancer Hospital of China Medical University, Shenyang, China; ^2^Department of Breast Surgery, The First Affiliated Hospital of China Medical University, Shenyang, China; ^3^National Cancer Center/National Clinical Research Center for Cancer/Cancer Hospital, Chinese Academy of Medical Sciences and Peking Union Medical College, Beijing, China

**Keywords:** Ganglioside monosialic acid, capecitabine, chemotherapy-induced peripheral neurotoxicity, metastatic breast cancer, Utidelone

## Abstract

**Purpose:**

This study aimed to assess the efficacy of utidelone, a novel genetically engineered epothilone analog, combined with capecitabine in our center and, furthermore, to identify whether ganglioside monosialic acid (GM1) improved chemotherapy-induced peripheral neurotoxicity (CIPN).

**Methods:**

Fifty-five eligible female patients with metastatic breast cancer were enrolled in our single-center phase III BG01-1323L trial. Utidelone combined with capecitabine-induced peripheral neuropathy was analyzed, and susceptible genes were detected in a germline panel by next-generation sequencing (NGS).

**Results:**

In our single-center study, median progression-free survival and overall survival (OS) improved in the utidelone plus capecitabine group (mPFS: 238 vs. 189 days, *P* = 0.263; OS: 20.9 vs. 12.9 months, *P* = 0.326). The median time to severe CIPN reported was 29 days in grade 1, 49 days in grade 2, and 103 days in grade 3. Greatly longer improvement time was indicated in grade 1 (77 vs. 20 days in grade 2, 13 days in grade 3). In the combined group, 19 patients with G2 or G3 CIPN were assigned to the GM1 group and 9 patients to the control group. After intervention, the GM1 group was reported to demonstrate a statistically lower incidence of grade 3 CIPN [GM1 group: 1 of 19 (5.3%); control group: 4 of 9 (44.4%), *P* = 0.026]. However, there were no statistically significant differences in germline single nucleotide polymorphism (SNP) between grade 3 and grade 1 CIPN cohorts.

**Conclusion:**

Ganglioside monosialic acid potentially decreases severe utidelone plus capecitabine-induced peripheral neuropathy in metastatic breast cancer, and further investigation is needed to validate the manageable efficacy of GM1 in CIPN.

**Clinical Trial Registration:**

ClinicalTrials.gov, identifier NCT02253459.

## Background

Breast cancer accounted for 25% of all newly diagnosed cancer worldwide and 15% of all newly diagnosed cancer in females in China (1). Despite advances in target therapy, endocrine therapy, and immunotherapy, chemotherapy remains the fundamental strategy for breast cancer patients. The standard adjuvant chemotherapy significantly improved disease-free survival (DFS) and overall survival (OS) in patients with early breast cancer, especially anthracyclines and taxanes (2). However, above 30% of early breast cancer relapses or distant metastasis contributed to drug resistance in a metastatic setting, and this has led to an increase in heavy pretreatment with and resistance to anthracyclines and taxanes. Some metastatic breast cancer patients are limited to anthracyclines due to the risk of cumulative cardiac-related toxicities. Recently, several novel chemotherapies have been approved for patients with metastatic breast cancer, including gemcitabine, capecitabine, and eribulin, especially for metastatic triple-negative breast cancer (3).

With limitations related to cost and availability of drugs for recurrent breast cancer in China, we need novel chemo-drugs as monotherapy or combined therapy to improve the survival of patients with previously treated locally recurrent or metastatic breast cancer. Patients with taxane resistance remain sensitive to epothilones due to the different molecular structure and targets. Utidelone is a novel genetically engineered epothilone analog by genetically manipulating the biosynthetic gene cluster in *Sorangium cellulosum*. Utidelone has also been shown to have clinically manageable toxicities in phase I and has a median progression-free survival (PFS) of 7.9 months and an objective response ratio (ORR) of 42.4% combined with capecitabine in phase II (4). In the BG01-1323L study (phase III, randomized, NCT02253459), adding utidelone to capecitabine showed significantly improved PFS and OS in metastatic breast cancer patients heavily treated with anthracycline and taxane. The median PFS was 8.44 months in the utidelone combined with capecitabine arm and 4.24 months in the capecitabine monotherapy arm {hazard ratio (HR) 0.46 [95% confidence interval (CI) 0.36, 0.59]} at the primary analysis (5), and the OS benefit resulted in a significant 32% reduction in risk of death (21.30 vs. 15.90 months, HR 0.68; 95% CI 0.52, 0.83, and *P* = 0.0024) at the confirmatory OS analysis (Xu B et al., in review).

Ixabepilone, as a similar epothilone analog like utidelone, has been approved by the FDA as a monotherapy in patients with previously treated advanced breast cancer, a progressive disease previously treated with anthracycline, taxane, and capecitabine (6). As we all know, serious adverse effects related to ixabepilone affected its global application for metastatic breast cancer, such as serious myelosuppression and fatigue, which led to discontinuation of ixabepilone, and it was shown that utidelone was less costly and well-tolerable than ixabepilone treatment (7, 8). In the BG01-1323L study (5), patients received a median of six cycles of treatment in the utidelone combination with capecitabine cohort (range 1–34 cycles) and also a median of six cycles in the capecitabine monotherapy cohort (1–24 cycles). The main common adverse event related to utidelone was grade 3 peripheral neuropathy (22% in the combination cohort and <1% in the monotherapy cohort), which resulted in a dose reduction or discontinuation of utidelone. However, the utidelone-related toxicities were manageable and no grade 4 peripheral neuropathy was reported. This phase III trial verified and expanded on the efficacy and safety profiling of utidelone from 26 investigation sites in China, and most of the patients enrolled in this study were from our center (13.6%, 55/405). In our exploratory analysis, patients from our center experienced more peripheral neuropathy adverse events (which may have been related to more attention from investigators and patients in our center), than patients from other centers in China. We also prospectively assessed the efficacy of ganglioside monosialic acid (GM1) in the improvement of peripheral neuropathy in patients with G2 or G3 chemotherapy-related peripheral neuropathy.

Our study is a single-center analysis and investigative study that aims to assess the efficacy of utidelone combined with capecitabine in Chinese patients with metastatic breast cancer that had been pretreated with or developed resistance to anthracyclines or taxanes from the population of BG01-1312L and, furthermore, to verify the genomic landscape between G1 and G3 peripheral neuropathy and efficacy of GM1 on G2 or G3 peripheral neuropathy safety profiling in combination with utidelone and capecitabine.

## Methods

BG01-1323L is a phase III, randomized, open-label study conducted across 26 centers in China. The protocol was approved by Liaoning Cancer Hospital and Institute Ethics Committee and Institutional Review Board (#20140802-2). All participants provided written informed consent.

Female patients had metastatic breast cancer confirmed histologically or cytologically, with up to four prior chemotherapy therapy for the disease. Patients were eligible if they are 18 to 70 years of age, had one measurable disease at least assessed *via* imaging techniques, life expectancy of at least 3 months, and an Eastern Cooperative Oncology Group performance status (ECOG PS) of 0 or 1. Patients were excluded if they had received prior capecitabine treatment, if they had a history of peripheral neuropathy within 4 weeks before randomization of higher than grade 2 according to the Common Terminology Criteria for Adverse Events (CTCAE) version 4.03 (National Cancer Institute 2010), or if they had any other conditions that were not controlled and could affect the patient’s ability to comply with the study. To recruit patients to our trial, we first informed them that a trial was taking place and informed them about the possible benefits and side effects of the treatment and also the procedures of the clinical study and the intervention of chemotherapy-related peripheral neuropathy. If the patients agreed to participate in the study, they were then required to provide written informed consent.

### Procedures

Eligible patients were randomized (computer-generated by an independent randomization statistician and loaded into the Interactive Website Response System) 2:1 to utidelone (30 mg/m^2^ intravenously once per day on days 1–5 every 3 weeks, Beijing Biostar Technologies, Ltd. Beijing, China) plus capecitabine (1,000 mg/m^2^ twice per day orally on days 1–14 every 3 weeks, Hoffmann-La Roche AG, Basel, Switzerland) or capecitabine (1,250 mg/m^2^ twice per day orally on days 1–14 every 3 weeks, Hoffmann-La Roche AG, Basel, Switzerland). This trial was open label, and eligible patients, physicians, and individuals assessing outcomes and analyzing data were not masked to treatment allocation. Utidelone and capecitabine were given until disease progression or unacceptable toxicity. Dose reductions were permitted for utidelone or capecitabine to manage adverse effects. Protocol-specific guidance for utidelone or capecitabine dose reduction and discontinuation is provided in the Appendix (Supplementary Table 1).

### Assessments

The primary endpoint was independent radiology review committee-assessed PFS, defined as the time from randomization to the first occurrence of disease progression, or death from any cause after the last tumor assessment or prior to the first tumor assessment. Tumor assessments were conducted every 6 weeks for the first eight cycles and every 9 weeks from the ninth cycle until progression. Adverse events were continuously monitored throughout the study by an investigator and laboratory tests and were graded per CTCAE v4.03.

### Clinician-Reported Outcome Measures of Chemotherapy-Induced Peripheral Neurotoxicity

Chemotherapy-induced peripheral neuropathy was assessed by the clinician-reported outcome CTCAE, consisting of two sections relating to sensory neuropathy in the hands or feet in the previous 7 days. The first question assessed the severity of numbness and tingling in the hands and feet from 0 (none) to 4 (very severe) and the second assessed interference of numbness and tingling with “usual daily activities” from 0 (not at all) to 4 (very much). The questionnaires were added to the questionnaire toward the end of the recruitment period, and all patients accrued subsequently completed the questions with the help of research assistants. Peripheral neuropathy assessments were performed at screening, baseline, every 6 weeks from randomization until the treatment discontinuation visit (or more frequently as needed), every 6 months thereafter for the first year, and annually for up to 3 years until the end of the study.

### Intervention for Peripheral Neuropathy

Patients who experienced G2 or G3 chemotherapy-induced peripheral neurotoxicity (CIPN) in the utidelone plus capecitabine group were selected for this exploratory analysis, and the patients were assigned to the GM1 cohort or control cohort (no intervention). Utidelone-based treatment was administered the first day of each chemotherapy cycle. GM1 (80 mg) was administered at each course of utidelone plus capecitabine chemotherapy. Intravenous infusion was performed once per day for 5 days (day 0, day 1, day 2, day 3, and day 4) during each cycle of chemotherapy.

### Genomics Analysis

Retrospective metabolic enzyme single nucleotide polymorphism (SNP) testing of plasma was performed with next-generation sequencing (NGS; Illumina Seq 2000). The plasma was collected using 5 ml of blood. A total of 5 ml of blood was used for DNA extraction using DNA purification kit. DNA was eluted in 100 μl, 150 μl of which was used for SNP detection and germline variant mutation detection. SNP detection was performed for 11 genes, namely ABCB1, CDA, CYP19A1, CYP2D6, DPYD, ERCC1, GSTP1, NQO1, TP53, TYMS, and UGT1A1. The other DNA was studied to identify germline mutations by 398-gene panels (Berry oncology).

### Statistical Methods

In our single analysis, statistical testing is considered exploratory and safety analyses are descriptive. All results were recorded by electronic data capture system. The Kaplan–Meier approach was used to estimate median PFS for the utidelone combined with capecitabine cohort and the capecitabine-alone cohort. The two-sided stratified log-rank test was used to compare PFS between the two treatment arms. The corresponding HR was estimated using a Cox regression model. Analysis of PFS was done with a two-sided α level of 0.05. The Cox proportional hazards model, stratified by disease type (visceral vs. non-visceral disease) and peripheral neuropathy grades, was used to estimate the HR and its 95% CI. An estimate of the objective response rate and its 95% CI (Clopper–Pearson) were calculated for each treatment arm and the difference was calculated with 95% CIs (Hauck–Anderson).

## Results

### Study Population

**FIGURE 1:**
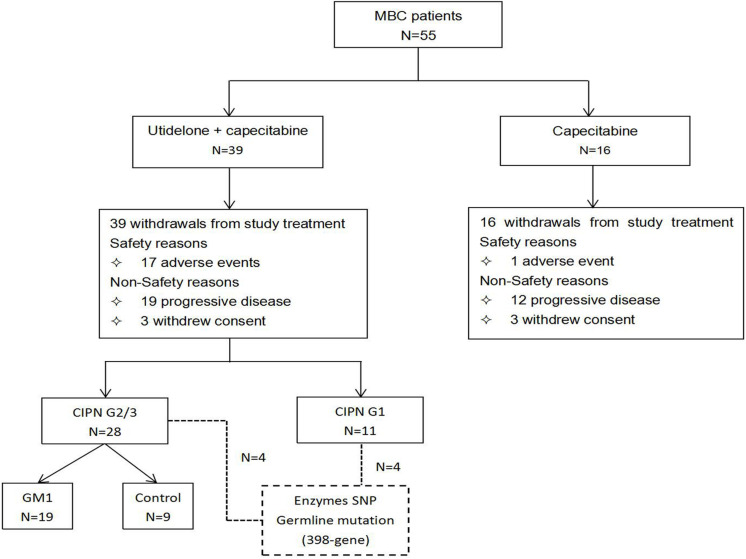
Patient disposition in our center population in the BG01-1323L study. Flow diagram showing patient enrollment, allocation, follow-up, and analysis in our center population from the BG01-1323L study.

During the period from October 11, 2014, to October 15, 2015, a total of 55 female patients were randomized in our center populations: 39 to the utidelone plus capecitabine arm and 16 to the capecitabine-alone arm (our center populations; Figure 1). All the patients received at least one cycle of treatment in both arms. The safety populations therefore comprised 39 and 16 patients in the utidelone plus capecitabine and capecitabine-alone arms, respectively. By the data cutoff date for PFS and OS analysis (December 17, 2018), the median follow-up was 626 days (95% CI 464–788 days) in the utidelone plus capecitabine arm and 388 days (95% CI 270–506 days) in the capecitabine-alone arm. The baseline demographics and disease characteristics were generally balanced between arms in our center and the total intention-to-treat (ITT) cohort (Table 1). At baseline, the majority of patients enrolled had visceral disease (82.1%, 93.8%), and 89.7 and 100% had ECOG PS of 1 in the combination and capecitabine-alone cohorts, respectively. Prior anti-HER2 therapy and previous endocrine therapy had been given in 2.6% and 76.9% of the enrolled patients in the combination cohort.

**TABLE 1:** Baseline patient demographics and characteristics for our center population and the total intention-to-treat population.

	Our center population		Total ITT population	
		
	Utidelone plus Capecitabine (*n* = 39)	Capecitabine alone (*n* = 16)	Utidelone plus Capecitabine (*n* = 270)	Capecitabine alone (*n* = 135)
Female sex, *n*(%)	39 (100%)	16 (100%)	270 (100%)	135 (100%)
**Age (years)** Median (range) <60 ≥60	52 (25–70) 32 (82.1%) 7 (17.9%)	51 (37–69) 15 (93.8%) 1 (6.2%)	50 (25–70) 237 (88%) 33 (12%)	50 (27–69) 117 (87%) 18 (13%)
**ECOG performance status** 0 1 2	4 (10.3%) 35 (89.7%) 0	0 16 (100.0%) 0	91 (34%) 171 (63%) 6 (2%)	39 (29%) 91 (67%) 4 (3%)
**HR and HER2 status** HR-positive, HER2-negative HR-positive, HER2-positive HR-negative, HER2-positive HR-negative, HER2-negative	21 (53.8%) 8 (20.5%) 4 (10.3%) 6 (15.4%)	6 (37.5%) 3 (18.8%) 4 (25.0%) 3 (18.8%)	121 (44.8%) 45 (16.7%) 45 (16.7%) 59 (21.8%)	56 (41.5%) 20 (14.8%) 28 (20.7%) 31 (23.0%)
**Previous anti-HER2 therapy** Yes No	1 (2.6%) 38 (97.4%)	0 16 (100.0%)	42 (15.6%) 228 (84.4%)	11 (8.1%) 124 (91.9%)
**Previous endocrine therapy** Yes No	30 (76.9%) 9 (23.1%)	8 (50.0%) 8 (50.0%)	147 (54.4%) 123 (45.6%)	72 (53.3%) 63 (46.7%)
**Previous chemotherapy regimens** Median (range) 1 2 ≥3	2 (1–5) 14 (35.9%) 15 (38.5%) 10 (25.6%)	2 (1–3) 4 (25.0%) 11 (68.8%) 1 (6.2%)	2 (1–6) 69 (26%) 95 (35%) 106 (39%)	2 (1–5) 29 (21%) 51 (38%) 55 (41%)
Previous capecitabine treatment	1 (2.6%)	0	28 (10%)	21 (16%)
**Tumor metastasis** Lymph node Lung Bone Liver Peritoneum Pleura Other*	21 (53.8%) 24 (61.5%) 24 (61.5%) 19 (48.7%) 1 (2.6%) 2 (5.1%) 9 (23.1%)	4 (25.0%) 8 (50.0%) 9 (56.3%) 11 (68.8%) 0 1 (6.2%) 7 (43.8%)	161 (60%) 145 (54%) 131 (49%) 123 (46%) 45 (17%) 26 (10%) 13 (5%)	79 (59%) 70 (52%) 76 (56%) 68 (50%) 21 (16%) 15 (11%) 18 (13%)
**Visceral metastases** Yes No	32 (82.1%) 7 (17.9%)	15 (93.8%) 1 (6.2%)	216 (80%) 53 (20%)	110 (81%) 24 (18%)
**Lymph node metastases** Yes No	21 (53.8%) 18 (46.2%)	4 (25.0%) 12 (75.0%)	167 (62%) 102 (38%)	79 (59%) 56 (41%)
**Number of metastatic sites** ≤2 >2	19 (48.7%) 20 (51.3%)	7 (43.7%) 9 (56.3%)	135 (50%) 134 (50%)	66 (49%) 68 (50%)

*^∗^Other sites of metastasis were neck (*n=1*), mediastinum (*n=1*) and scalp (*n=1*).*

### Progression-Free Survival and Response Rate

**FIGURE 2:**
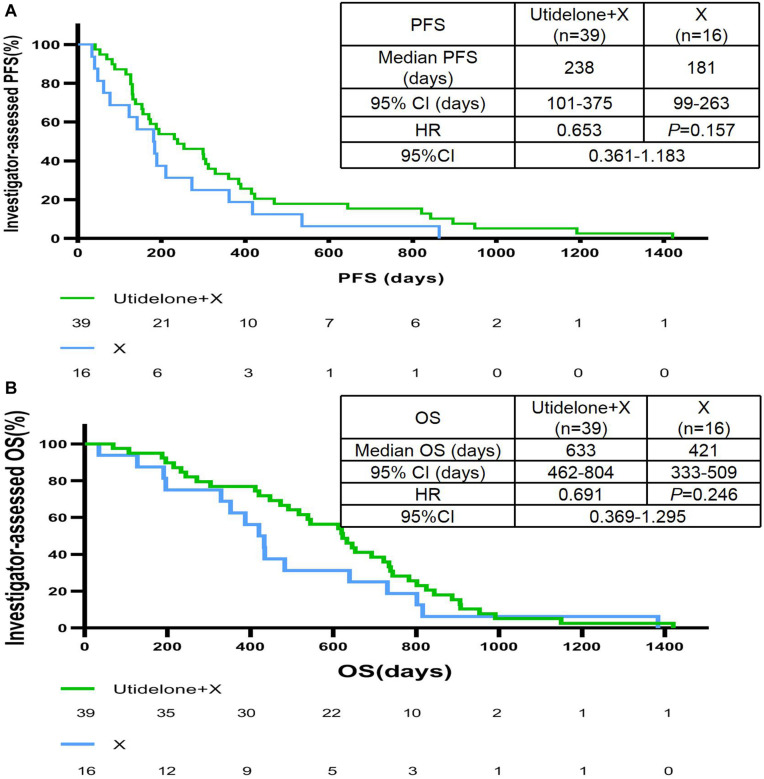
IRC-assessed PFS **(A)** and OS **(B)** in our center population. *CI*, confidence interval; *X* capecitabine, *ECOG PS*, Eastern Cooperative Oncology Group performance status; *ER*, estrogen receptor; *HER2*, human epidermal growth factor receptor 2; *HR*, hazard ratio; *mo*, months; *U*, utidelone; *PFS*, progression-free survival; *PgR*, progesterone receptor; *IRC*, independent radiology review committee; and *OS*, overall survival.

Treatment with utidelone plus capecitabine resulted in an improvement in independent radiology review committee-assessed PFS compared with capecitabine alone in our center, similar to the total ITT population. The median PFS was 238 days in the utidelone plus capecitabine arm (95% CI 96 days, 380 days) compared with 189 days in the capecitabine-alone arm (95% CI 114 days, 264 days). The HR was 0.74 (95% CI 0.41, 1.34, and *P* = 0.263; Figure 2A). Subgroup analyses were consistent with overall PFS results. Patients with measurable disease at baseline in the utidelone plus capecitabine arm achieved an objective response rate of 41.0% compared with 37.5% in the capecitabine-alone arm—only a difference of 3.5% (95% CI −0.4 to 7.4), achieving less than that in the total ITT population (a difference of 11.5%, 95% CI 3.7–19.3; Table 2). The proportion of patients who had a clinical benefit was higher in the utidelone plus capecitabine group than in the capecitabine-alone group among patients in our center population, and the absolute difference was 10.0% (53.8 vs. 43.8%), which was similar to that in the ITT population (49.4 vs. 34.4%; Table 2). OS events occurred in 34 patients in the utidelone plus capecitabine arm (94.4%) and 14 patients in the capecitabine-alone arm (87.5%). Figure 2B shows the final outcomes regarding OS. Prolonged OS was indicated in the group that received utidelone and capecitabine combination therapy: the median OS was 20.9 months in the utidelone and capecitabine group and 12.9 months in the capecitabine-alone group, respectively, (hazard ratio 0.69; 95% CI 0.37 to 1.30; *P* = 0.326 by the log-rank test).

**TABLE 2:** Objective response and clinical benefit rates in our center and ITT population.

	Out center population (*n* = 55)		Total ITT population (*n* = 405)	
		
	Utidelone + capecitabine (*n* = 39)	Capecitabine alone (*n* = 16)	Utidelone + capecitabine (*n* = 270)	Capecitabine alone (*n* = 135)
Objective response	41.0%	37.5%	39.6%	28.1%
	Difference 3.5% *P* = 0.808		Difference 11.5% *P* = 0.023	
Clinical benefit^†^	53.8%	43.8%	49.4%	34.4%
	Difference 10.0% *P* = 0.496		Difference 15.0% *P* = 0.004	
Complete response	0	0	2 (1%)	1 (1%)
Partial response	16 (41.0%)	6 (37.5%)	105 (39%)	37 (27%)
Stable disease	18 (46.2%)	6 (37.5%)	123 (46%)	59 (44%)
Progressive disease	1 (2.6%)	2 (12.5%)	27 (10%)	22 (16%)
Unavailable	4 (10.3%)	2 (12.5%)	13 (5%)	16 (12%)

*^†^Complete response plus partial response plus stable disease for 24 weeks.*

### Treatment Exposure and Dosage Reduction

The median numbers of utidelone and capecitabine cycles received by patients in our center population were 6.0 cycles of utidelone (range 1–41) and 6.0 cycles of capecitabine (range 1–64) in the utidelone plus capecitabine arm and 6.0 cycles of capecitabine (range 1–38) in the capecitabine-alone arm (1–32), as shown in Table 3. Patients received utidelone with a median total dose of 1,458.0 mg [interquartile range (IQR) 948.0–2,455.5 mg] and capecitabine with a median total dose of 21,000.0 mg (IQR 12,500.0–35,000.0 mg) in the utidelone plus capecitabine arm and capecitabine with a median total dose of 22,000.0 mg (IQR 14,000.0–46,375.0 mg) in the capecitabine-alone arm. Patients received utidelone and capecitabine treatment for median durations of 19.6 weeks (range 3.3–123.3 weeks) in the utidelone plus capecitabine arm. Patients received capecitabine treatment for median durations of 19.8 weeks (range 2.9–204.0 weeks) in the capecitabine-alone arm.

**TABLE 3:** Exposure and dosage reduction of chemotherapy in utidelone plus capecitabine and capecitabine-alone groups.

	Utidelone plus Capecitabine group		Capecitabine-alone group
		
	Utidelone	Capecitabine	Capecitabine
Number of patients	39	39	16
**Exposure cycles**			
Median (cycle)	6.0	6.0	6.0
Range (cycle)	(1–41)	(1–64)	(1–38)
**Exposure dosage**			
Median (mg)	1458.0	21000.0	22000.0
IQR (mg)	(948.0–2455.5)	(12500.0–35000.0)	(14000.0–46375.0)
**Dosage reduction**			
Once	41.0%	48.7%	31.2%
Twice	17.9%	12.8%	0
**Dosage reduction cycles**			
Median (cycle)	5.5	5	4
Range (cycle)	4–11	3–16	2–6
**Therapy duration**			
Median (week)	19.6		19.8
Range (week)	3.3–123.3		2.9–204.0
Discontinuation rate	43.6%		6.3%
	χ^2^ = 7.37, *P* = 0.007		

*IQR, Interquartile range.*

The dose of utidelone was reduced according to protocol in 16 of the 39 patients (41.0%) and the dose of utidelone was reduced twice in 7 of 39 patients (17.9%), the dose of capecitabine was reduced in 19 of the 39 patients (48.7%) and the dose of capecitabine was reduced twice in 5 of the 39 patients (12.8%) in the combination group, whereas the matching capecitabine was reduced in 5 of the 16 patients (31.2%) in the capecitabine-alone group (Table 3). The median dose reduction cycles and the median numbers of cycles to discontinuation for both arms are shown in Table 3. The median time to the first reduction of utidelone was 5.5 cycles (range 4–11 cycles) and the median time to the second reduction of utidelone was 7.0 cycles (range 6–12 cycles). The median time to the first and second reductions of capecitabine were 5 cycles (range 3–16 cycles) and 10 cycles (range 7–24 cycles) in the combination group, respectively. In the capecitabine-alone cohort, the median time to reduction was 4 cycles (2–6 cycles) and no second reduction was found. The main reason for the permanent discontinuation of the study treatment was disease progression in our center population, which occurred in 19 patients (48.7%) in the utidelone plus capecitabine group and in 12 patients (75.0%) in the capecitabine-alone group. Overall permanent discontinuation of study treatment as a result of adverse events (including chemotherapy-induced peripheral neuropathy) occurred in 17 patients (43.6%) in the utidelone plus capecitabine group (utidelone or capecitabine or both) and in 1 patient (6.3%) in the capecitabine-alone group (Table 3). Patients had permanent discontinuation due to adverse events in our center population more than that in the total ITT population (43.6 vs. 23.2%, *χ*^2^ = 7.37, and *P* = 0.007).

### Clinician-Graded Assessed Chemotherapy-Induced Peripheral Neurotoxicity

**FIGURE 3:**
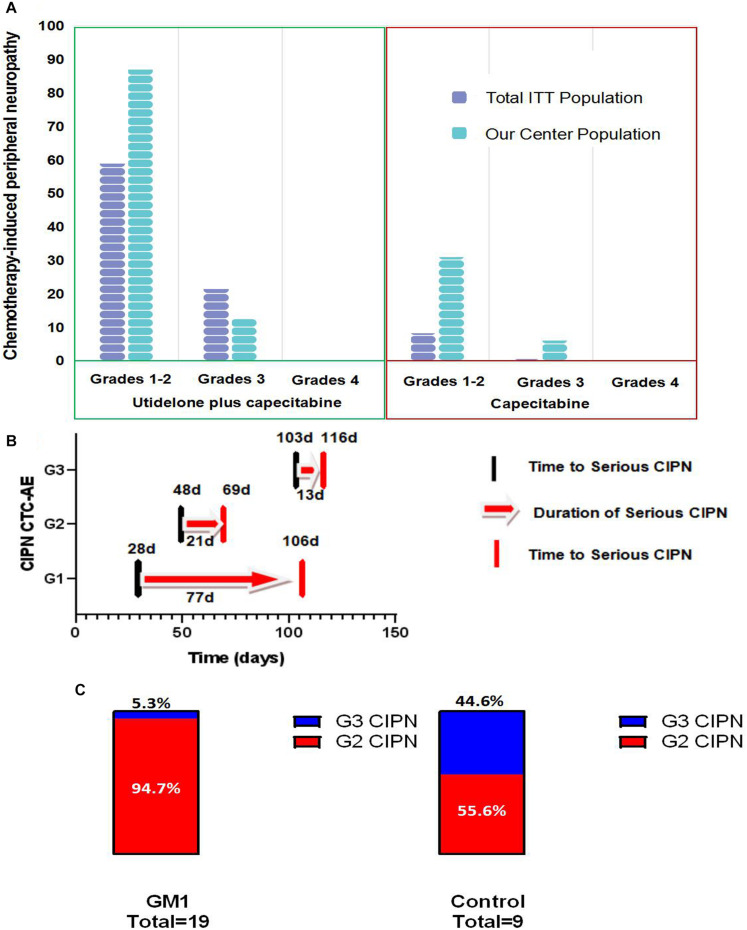
Bar chart of patient-reported chemotherapy-induced peripheral neuropathy in total population and our center population **(A)**, the time of appearance, remission and duration of serious CIPN **(B)** bar chart of patient-reported grade 2 and grade 3 of CIPN in GM1 and control groups **(C)**.

Figure 3 illustrates the maximum clinician-reported CIPN scores at any time during the BG01-1323L study in our center population and total ITT population. Overall, 100% of patients (39 patients) rated their CIPN toxicity in the utidelone plus capecitabine group as mild (grade 1, 11 patients), moderate (grade 2, 23 patients), and severe (grade 3, 5 patients) according to CTCAE v4.03, and none reported grade 4 in our center population. Approximately, 37.5% of patients (6 patients) rated their CIPN toxicity in the capecitabine-alone group as grade 2 (5 patients) and grade 3 (1 patient). Patients in the capecitabine-alone group reported more moderate (grade 1/grade 2) and severe chemotherapy-induced peripheral neuropathy (grade 3) in our center population than those in the total ITT population (G1/2: 31.2% in our center population vs. 8.4% in total ITT population; G3: 6.2% in our center population vs. 0.8% in total ITT population). Similarly, of the patients treated with utidelone plus capecitabine, 89.7% (35 patients) reported moderate chemotherapy-induced peripheral neuropathy in our center population, more than those in the total ITT population (G1/2: 60.3% in the total ITT population). However, less patients in the utidelone plus capecitabine group reported severe CIPN in our center population (G3: 12.8% in our center population vs. 25.1% in the total ITT population, *P* = 0.09). Reasons for less severe CIPN in our center population may be the intervention of GM1 to improve neuropathy in the utidelone plus capecitabine group. No substantial differences were observed for the median time to grade 2 or worse neurotoxicity when CIPN was measured by the NCI-CTCAE. The median time to severe CIPN reported was 28.0 days in grade 1 (IQR 6.0∼48.0 days), 48.0 days in grade 2 (IQR 23.0∼111.3 days), and 103.0 days in grade 3 (IQR 68.8∼119.0 days). The median improvement time (from G3 to G2/G1, from G2 to G1/G0, from G1 to G0) was 77.0 days in grade 1 (IQR 33.0∼167.0 days), 21.0 days in grade 2 (IQR 10.8∼77.5 days), and 13.0 days in grade 3 (IQR 3.5∼27.5 days).

In the combined group, 19 patients with G2 or G3 CIPN were assigned to the GM1 group and 9 patients to the control group. After the intervention, the GM1 group was reported to demonstrate a statistically lower incidence of grade 3 CIPN [GM1 group: 1 of 19 (5.3%); control group: 4 of 9 (44.4%), Fisher’s exact test, *P* = 0.026, Figure 3C]. *Post hoc* analyses showed there was no statistically significant difference between the GM1 group and the control group for permanent discontinuation [8 of 19 (42.1%) vs. 6 of 9 (66.7%), *and P* = 0.42].

### Evaluation of Chemotherapy-Induced Peripheral Neurotoxicity and Survival

**FIGURE 4:**
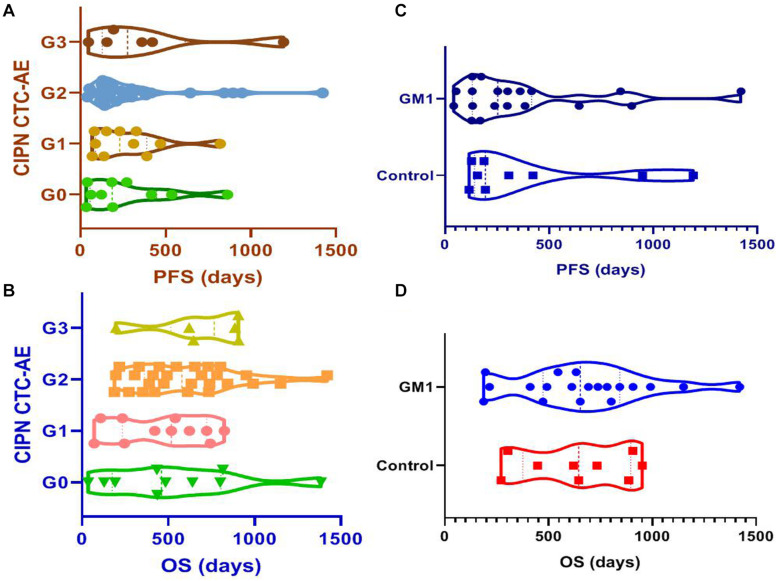
IRC-assessed PFS among patients with diverse grades of CIPN **(A)**, IRC-assessed PFS between GM1 and control groups **(B)**, IRC-assessed OS among patients with diverse grades of CIPN **(C)**, IRC-assessed OS between GM1 and control groups **(D)**.

Results suggested that patients who received utidelone plus capecitabine experienced moderate and severe CIPN (the same benefit from chemotherapy) than those with grade 1 CIPN or none CIPN [G0/G1 vs. G2/G3: median PFS = 189.0 vs. 194.0 days, HR = 0.79, 95% CI 0.45∼1.38, *P* = 0.415; G0: median PFS = 186.5 days (IQR 56.5∼446.5 days); G1: median PFS = 231.0 days (IQR 88.0∼389.0 days); G2: median PFS = 198.5 days (IQR 130.0∼378.3 days); and G3: median PFS = 277.0 days (IQR 128.0∼614.5 days); Figure 4A]. Likewise, the observed median OS of the capecitabine-alone group was 620.0 days in moderate and severe CIPN and 483.0 days in patients who experienced G1 or none CIPN [G0/G1 vs. G2/G3: median OS = 483.0 vs. 620.0 days, HR = 0.74, 0.41∼1.35, *P* = 0.335; G0: median OS = 459.0 days (IQR 174.3∼804.5 days); G1: median OS = 517.0 days (IQR 233.0∼722.0 days); G2: median OS = 578.5 days (IQR 361.0∼771.0 days); and G3: median OS = 765.5 days (IQR 514.0∼906.3 days); Figure 4B]. In the utidelone plus capecitabine cohort, patients who received GM1 intervention benefit more from chemotherapy than patients without intervention for CIPN (median PFS: 253 vs. 194 days, HR = 0.81, 95% CI 0.35–1.89, *P* = 0.634; Figure 4C) and there was similar OS between both the GM1 group and the control group (median OS: 653 vs. 645 days, HR = 1.21, 95% CI 0.50–2.89, *P* = 0.667; Figure 4D).

### Metabolic Enzyme Single Nucleotide Polymorphism Landscape for Chemotherapy-Induced Peripheral Neurotoxicity

Although the study was powered to detect differences between treatment groups as main effects only, in exploratory analyses, we examined the potential interaction between CIPN grades and genomics landscapes. Eight patients were selected for genomics analysis from the combined group and did not receive GM1 to avoid interference with intervention. The patients were grouped as follows: four patients who experienced mild CIPN (grade 1) and four patients with severe CIPN (grade 3). The results of the exploratory responder analysis revealed no difference of SNP in 11 metabolic enzymes (ABCB1, CDA, CYP19A1, CYP2D6, DPYD, ERCC1, GSTP1, NQO1, TP53, TYMS, and UGT1A1), as shown in Table 4. We also concurrently evaluated germline variants and there were no statistically significant pathogenic germline variants related with severe neuropathy toxicity. One patient with severe CIPN (G3-01) indicated BRCA1 pathogenic variant (exon10, c.3296delC, p. P1099Lfs) and ATM variants (p. V1671A and p.S2707C). Patients with G3 of CIPN harbored AXIN2 (Patient G3-02:exon6, c.A1235T, p.N412I), FANCM (Patient G3-03: exon14, c.A3308C, p. H1103P), FANCC (Patient G3-03: exon9, c.C854A, p.A285D), CBL (Patient G3-03: exon11, c.A1665G, p. G555G), SMARCA4 (Patient G3-04: exon4, c.A602T, p. Q201L) and FH (Patient G3-04: exon8, c.A1195G, p. S399G). In patients with G1 of CIPN, two patients was reported as negative in pathogenic or uncertain significance mutations (Patient G1-07/08), and SDHD (Patient G1-05: exon3, c.A217G, p. S73G), BRAF (Patient G1-05: exon18, c.G2156A, p. R719H), FH (Patient G1-05: exon2, c.G1936A, p. D65N), MLH3 (Patient G1-06: exon9, c.G3950A, p. R1317Q) and ESR1 (Patient G1-06: exon3, c.G652A, p. D218N) were identified in other patients with CIPN by G1. Due to this small sample, we further have to identify the potential correlation between germline variants and severe neuropathy toxicity in large patients treated with utidelone and capecitabine.

**TABLE 4:** Correlation between severe chemotherapy-induced peripheral neuropathy and metabolic enzymic SNPs.

Genes		ABCB1	CDA	CYP19A1		CYP2D6	DPYD	ERCC1	GSTP1	NQO1	TP53	TYMS	UGT1A1
Duration Time	CIPN	rs1045642	rs2072671	rs2236722	rs4646	rs3892097	rs3918290	rs11615	rs1695	rs1800566	rs1042522	rs151264360	rs8175347
26	G3	AG	AA	AA	AC	CC	CC	GG	AA	AA	CG	S-del	6/6
11	G3	GG	AA	AA	CC	CC	CC	GG	AA	AG	CG	S-del	6/7
4	G3	GG	AA	AA	AC	CC	CC	GG	AA	GG	GG	TT	6/7
16	G3	AG	AA	AA	CC	CC	CC	AG	AA	AA	CG	S-del	6/7
16	G1	GG	AC	AA	CC	CC	CC	GG	AA	AG	CG	S-del	6/6
84	G1	GG	AA	AG	AC	CC	CC	GG	AA	GG	CG	D-del	6/7
145	G1	AG	AA	AA	AC	CC	CC	GG	AA	AG	GG	TT	6/6
21	G1	GG	AC	AA	AA	CC	CC	GG	AA	GG	GG	S-del	6/6

*del: del/del; S-del:TTAAAG/del; TT: TTAAAG/TTAAAG; 66: (TA)6/(TA)6; and 67:(TA)6/(TA)7.*

## Discussion

Our center population from the BG01-1323L study suggested superiority in efficacy data and demonstrated consistency with the total ITT results from this study (5). Utidelone plus capecitabine prolonged PFS and OS in patients with metastatic breast cancer refractory to anthracycline and taxane in our center population, with a clinically meaningful benefit corresponding to a 26% reduction in the risk of disease progression [HR 0.74 (95% CI 0.41, 1.34)] and a 31% reduction in the risk of death [HR 0.69 (95% CI 0.37, 1.30)]. The treatment effect of OS was consistent with patients in the total ITT population. In the total ITT population in the BG01-1323L study, the HR for the statistically meaningful improvement of OS with the addition of utidelone to capecitabine was 0.68 (95% CI 0.52, 0.87) (5). However, it should be noted that differences exist between our center population and the total ITT population in the BG01-1323L study. Specifically, in terms of objective response rate and clinical benefit rate, the utidelone plus capecitabine combined and capecitabine-alone arms both demonstrated higher absolute rate in our center population. Patients with measurable disease at baseline in the utidelone plus capecitabine arm achieved an objective response rate of 41.0% compared with 37.5% in the capecitabine-alone arm—only a difference of 3.5% (95% CI −0.4 to 7.4), achieving less than that in total ITT population (39.6 vs. 28.1%, a difference of 11.5%, 95% CI 3.7–19.3) (5). The proportion of patients who had a clinical benefit was higher in the utidelone plus capecitabine group (53.8 vs. 43.8%), which was similar to the ITT population (49.4 vs. 34.4%). The difference may be correlated with the proportion of patients with more than three previous chemotherapy regimens in our center population being less than that in the total ITT population (25.6/6.2 vs. 39%/41% in the total ITT population). In our center population, favorable efficacy with utidelone was also observed in terms of PFS, which was longer in the utidelone arm compared with the capecitabine arm. This was also similar to what was observed in the total ITT population: the reduction of disease progression was slightly lower than that in the ITT population (26% in our center population and 54% in the total ITT population).

As would be expected based on previous studies with utidelone alone or utidelone plus capecitabine in metastatic settings (4, 5, 7, 8), utidelone improved the survival for metastatic or locally advanced breast cancer patients previously treated with anthracycline and taxane. Utidelone, as an epothilone analog, remains sensitive to the tumor with anthracycline or taxane resistance. Likewise, ixabepilone, a semisynthetic epothilone analog, has been approved by the FDA as a monotherapy or combination therapy with capecitabine for patients with metastatic or locally advanced breast cancer, especially those who have anthracycline, taxane, or capecitabine resistance (6). However, ixabepilone-related toxicities always resulted in permanent discontinuation, due to myelosuppression, peripheral neuropathy, and fatigue (9). In our center population, the incidence of peripheral neuropathy after utidelone treatment decreased upon cessation of capecitabine. Severe peripheral neuropathy occurred more frequently in the combined chemotherapy vs. capecitabine-alone regimen.

Our center analysis demonstrated that the treatment effect was consistent for most of the patients experiencing peripheral neuropathy. The combination of utidelone and capecitabine was tolerable in our center and total ITT populations. The overall safety profile was consistent with that reported in the BG01-1323L study. However, compared with the capecitabine-alone arm, the addition of utidelone to capecitabine significantly increased the incidence of grade 3 peripheral neuropathy and resulted in more dose reduction of chemotherapy. Despite a higher rate of patients with permanent discontinuation of chemotherapy, patients remain to benefit more from the combination of utidelone and capecitabine. We speculated that this phenomenon was due to the superior efficacy of utidelone in combination with capecitabine. Previous studies suggested that capecitabine was correlated frequently with peripheral neuropathy, and utidelone as an epothilone analog produced taxane-like peripheral neuropathy (10, 11). If we exchange capecitabine with gemitabine or other mechanism drugs, we speculated that patients may get similar efficacy but less effect on peripheral neuropathy, and further investigation is needed to develop different combinations of drugs with utidelone for metastatic breast cancer treatment. Importantly, our investigative analysis revealed that GM1 improved the symptom score of peripheral neuropathy and significantly decreased severe peripheral neuropathy. Because of the limited application of GM1 only in patients with grade 2 or worse peripheral neuropathy in this study, this makes the efficacy of GM1 only for decreasing the ratio of patients with grade 3 peripheral neuropathy, rather than the ratio of all grades of peripheral neuropathy. The usage cycle of GM1 was in advance to prevent chemotherapy-induced neurotoxicity and mostly exhibited more potential efficacy. Notably, GM1, a kind of glycosphingolipid, was reported to effectively decrease the incidence of cumulative grade 3 oxaliplatin- and taxane-induced neurotoxicity in a non-randomized and retrospective study. However, no effect on acute, cold-induced neurotoxicity was found (12, 13). No substantial differences in permanent discontinuation of chemotherapy and effects on survival were noted between GM1 and control. In a phase III randomized, placebo-controlled, double-blind study, there were no significant differences between the GM1 and control arms with regard to the prevention of chronic neurotoxicity based on all the evaluation endpoints in patients with CRC (14). Interestingly, the trial showed that patients in the GM1 group were less troubled by acute neuropathy, with fewer patients in the GM1 group than in the placebo group reporting cold sensitivity and muscle cramps in patients with breast cancer (15). Comparing the results from these trials may be difficult or impossible because of the use of different dosages and regimens of treatment and the lack of standardization of the methods used in evaluating the extent or the incidence of neurotoxicity.

The mechanism underlying utidelone-induced peripheral neuropathy remains unclear. Utidelone and capecitabine may be associated with damage to dorsal root ganglion (DRG) neurons *via* energy failure or transport deficits, similar to oxaliplatin and taxane. Some studies suggested that oxaliplatin and taxane were associated with a dose-dependent reduction in plasma nerve growth factor (NGF), an essential mediator of the activity of DRG neurons (16–21). GM1 was approved as a neuroprotective agent against excitotoxic agents and ischemia with the mechanism of modulating neuronal plasticity and memory formation. Recently, several studies revealed that GM1 can significantly reduce the incidence of chemotherapy-induced peripheral neuropathy in colon cancer and breast cancer (12, 15).

This study has several limitations. Firstly, the sample of patients from the BG01-1323L study was limited to reveal the significant difference between the utidelone plus capecitabine and capecitabine-alone arms. Utidelone was received for approval in China, and further real-world investigation was designed to compare the efficacy of utidelone plus capecitabine and other drugs. Second, it was planned that patients will receive GM1 80 mg per day from day 0 to day 4 during chemotherapy in our study mainly based on convenience of clinical use. We did not perform a prior study on the different dosages and durations of GM1 to prevent utidelone- and capecitabine-induced peripheral neuropathy. Also, the optimal dose and duration of GM1 therapy is still unclear. Third, this study did not shed light on the precise mechanisms of utidelone-related peripheral neuropathy and the improvement GM1 has on neurotoxicity and its potential molecular mechanism. The plasma NGF and exosome-related NGF will be detected in patients with diverse chemotherapy-induced peripheral neuropathy in our center population in BG01-1323L and in patients in the real-world experience of utidelone. Last but not least, due to the limited number of patients for genomics analysis, no significant SNP and genomics mutation was found to be associated with chemotherapy-induced peripheral neuropathy. This may be due to an increased awareness of the established safety profile of these treatment regimens, resulting in overall better management.

## Conclusion

In summary, utidelone plus capecitabine was more efficacious compared with capecitabine alone for PFS with mild toxicity except for CIPN. These results reinforce the existing large body of evidence for utidelone plus capecitabine treatment of metastatic breast cancer and support the favorable benefit–risk profile of utidelone-based regimen in patients in the metastatic setting. Less grade 3 CIPN was assessed in our single center with GM1, and further investigation is needed to validate the manageable efficacy of GM1 to prevent CIPN and genomics landscape in patients treated with utidelone plus capecitabine, as an effective option for patients with metastatic breast cancer.

## Data Availability Statement

The original contributions presented in the study are publicly available. This data can be found here: the NCBI BioProject, accession number: PRJNA662155 (https://www.ncbi.nlm.nih.gov/bioproject/PRJNA662155).

## Ethics Statement

The studies involving human participants were reviewed and approved by Liaoning Cancer Hospital and Institute Ethics Committee. The patients/participants provided their written informed consent to participate in this study. Written informed consent was obtained from the individual(s) for the publication of any potentially identifiable images or data included in this article.

## Author Contributions

JX contributed to analyze the data and drafting of the work. JJ contributed to analyze the data. BX and TS designed the work and provided substantial contributions to the conception, all authors contributed to revising it critically for important intellectual content and read and approved the final the version of the manuscript to be published. All authors agreed to be accountable for all aspects of the work in ensuring that questions related to the accuracy or integrity of any part of the work are appropriately investigated and resolved. All authors contributed to the acquisition, analysis, or interpretation of data for the work.

## Conflict of Interest

The authors declare that the research was conducted in the absence of any commercial or financial relationships that could be construed as a potential conflict of interest.

## Supplementary Material

Supplementary Table 1 Dose modification for peripheral neuropathy toxicity.

## Abbreviations

CI: confidence interval
ECOG PS: Eastern Cooperative Oncology Group performance status
EC: ethics committee
ER: estrogen receptor
HER2: human epidermal growth factor receptor 2
HR: hazard ratio
OS: overall survival
PFS: progression-free survival
PgR: progesterone receptor
RECIST: Response Evaluation Criteria in Solid Tumors.
