# Supplementary material for: Ganglioside Monosialic Acid Alleviates Peripheral Neuropathy Induced by Utidelone Plus Capecitabine in Metastatic Breast Cancer From a Phase III Clinical Trial
Source: Front Oncol. 2020 Oct 9;10:524223. doi: 10.3389/fonc.2020.524223 (PMC7581989; doi:10.3389/fonc.2020.524223)
Supplement: Supplementary Table 1 — Dose modification for peripheral neuropathy toxicity. [file Data_Sheet_1.DOCX]

**Table S1. Dose modification for peripheral neuropathy toxicity**

| **Toxicity** | **Combination therapy arm** | | **Monotherapy arm** |
| --- | --- | --- | --- |
|  | **UTD1** | **Capecitabine** | **Capecitabine** |
| Grade 1PN | No change | No change | No change |
| Grade 2 PN | No change; 2nd and 3rd appearance, decrease 1 dose level † | No change; 3rd and 4th appearance, decrease 1 dose level † | Interrupt until resolved to ≤grade 1; 2nd and 3rd appearance, decrease 1 dose level |
| Grade 3 PN | Decrease 1 dose level; 2nd appearance, decrease 2 dose levels;  3rd appearance, discontinue utidelone§ | No change; 2nd and 3rd appearance, decrease 1 dose level † | Decrease 1 dose level; 2nd appearance, decrease 2 dose levels; 3rd appearance, discontinue capecitabine § |
| Grade 4 | Discontinue utidelone | Discontinue capecitabine | Discontinue capecitabine |

†Delay until toxicity resolved to ≤grade 1.

§If partial response or better is obtained, for the benefit of the patients, 50% of the original dose level can be used upon interruption and resolution to ≤ grade1.

PN=peripheral neuropathy. UTD1=utidelone.
